# Research on the application of distinguishing between benign and malignant breast nodules using MRI and US radiomics

**DOI:** 10.3389/fonc.2025.1630583

**Published:** 2025-07-16

**Authors:** Yifan Liu, Dan Zhou, Jing Liu, Jinding Wei, Xiao Hu, Xiaoli Yu

**Affiliations:** ^1^ Department of Radiology, Chongqing University Fuling Hospital, Chongqing, China; ^2^ Department of Pathology, Chongqing University Fuling Hospital, Chongqing, China; ^3^ Department of Ultrasound, Chongqing University Fuling Hospital, Chongqing, China

**Keywords:** breast, benign and malignant nodules, radiomics, machine learning, magnetic resonance imaging, ultrasound

## Abstract

**Objective:**

This study aims to develop and validate a model based on clinical and radiomic features to investigate its value in distinguishing between benign and malignant breast nodules.

**Methods:**

The study included 139 patients with breast diseases, divided into a training set (n=111) and a validation set (n=28) at an 8:2 ratio. All patients’ dynamic contrast-enhanced MRI (DCE-MRI), diffusion-weighted imaging (DWI), T1-weighted imaging (T1WI), T2-weighted imaging (T2WI), and ultrasound (US) images were uploaded to the 3D Slicer software. Using a double-blind method, regions of interest (ROIs) were manually delineated on T1WI, T2WI, DWI, the first phase of DCE, and US images. Radiomic models were constructed using radiomic features. A comprehensive model was built by combining clinical and radiomic features through multivariate logistic regression and visualized as a nomogram. The area under the curve (AUC), accuracy, specificity, and sensitivity of five different radiomic models were compared to evaluate their discriminatory performance. A combined model was created using the T2WI radiomic model and clinical features, and the predictive performance of the clinical model, radiomic model, and combined model were compared and validated.

**Results:**

For the T1WI radiomic model, the AUC values for the training and test sets were 0.885 and 0.778, respectively. For the T2WI radiomic model, the AUC values were 0.950 and 0.871. For the DCE radiomic model, the AUC values were 0.854 and 0.749. For the DWI radiomic model, the AUC values were 0.878 and 0.763. For the US radiomic model, the AUC values were 0.878 and 0.737. The combined model using T2WI and clinical features achieved AUC values of 0.975 and 0.942 for the training and test sets, respectively.

**Conclusion:**

The model combining T2WI and clinical features demonstrated higher value in non-invasively distinguishing between benign and malignant breast nodules.

Breast diseases represent a significant health concern for women globally, with breast cancer being one of the primary causes of cancer-related mortality among women. Advances in early screening and treatment technologies have improved survival rates for breast cancer patients, but it remains a major public health challenge. According to the 《2022 Global Cancer Statistics Report》, breast cancer has the highest incidence rate (11.6%) and mortality rate (6.9%) among all malignant tumors in women globally (1).Early detection and diagnosis of lesions can reduce breast cancer mortality (2, 3), Currently, the gold standard for breast cancer diagnosis in clinical practice is pathological biopsy (4), However, pathological tissue biopsy is an invasive procedure, and it confirms malignancy in less than 30% of breast tumors (5),Moreover, it carries risks of bleeding, tissue damage, and infection, with low repeatability. Traditional imaging methods for breast diseases primarily include mammography (mammogram), breast ultrasound, and breast magnetic resonance imaging (MRI) (6), These are tools of image informatics, demonstrating high levels of precision, sensitivity, and accuracy (7).Mammography can reduce breast disease mortality and is the most basic examination method for breast diseases, but it has poor visualization of lesions near the chest wall and within dense glandular tissue. Breast ultrasound has a lower detection rate for microcalcifications and relatively low specificity, and it requires skilled technicians to perform (3).Breast MRI without contrast has lower specificity compared to mammography and ultrasound, with overlapping features between benign and malignant lesions, but it has high sensitivity. Contrast-enhanced breast MRI can also provide hemodynamic information about the lesions, which is beneficial for qualitative diagnosis (8). In recent years, diffusion-weighted imaging (DWI) and dynamic contrast-enhanced MRI (DCE-MRI) have been used for the precise evaluation of breast lesions (9, 10), However, for BIRADS 3–4 category nodules, the assessment still largely depends on the subjective judgment of the observer (11). Most solid tumors exhibit high heterogeneity at the molecular phenotype, physiological, and genomic levels (12, 13), Radiomics can utilize various imaging data, such as CT, MRI, and US, employing advanced computational methods and machine learning techniques to analyze disease-related biological features, molecular markers, and clinical-pathological information. It extracts features that are not visible to the naked eye from images, using these features to assess the benign or malignant nature of lesions (14, 15), Therefore, this study applied various machine learning algorithms to develop and validate MRI and US radiomics models. By utilizing features from ultrasound images and multi-sequence MRI images of breast lesions and integrating clinical characteristics, a new radiomics predictive model was constructed and validated. The aim is to provide a simple and efficient diagnostic tool for the clinical assessment of benign and malignant breast lesions.

## 1 Study subjects and methods

### 1.1 Study subjects

**Figure 1:**
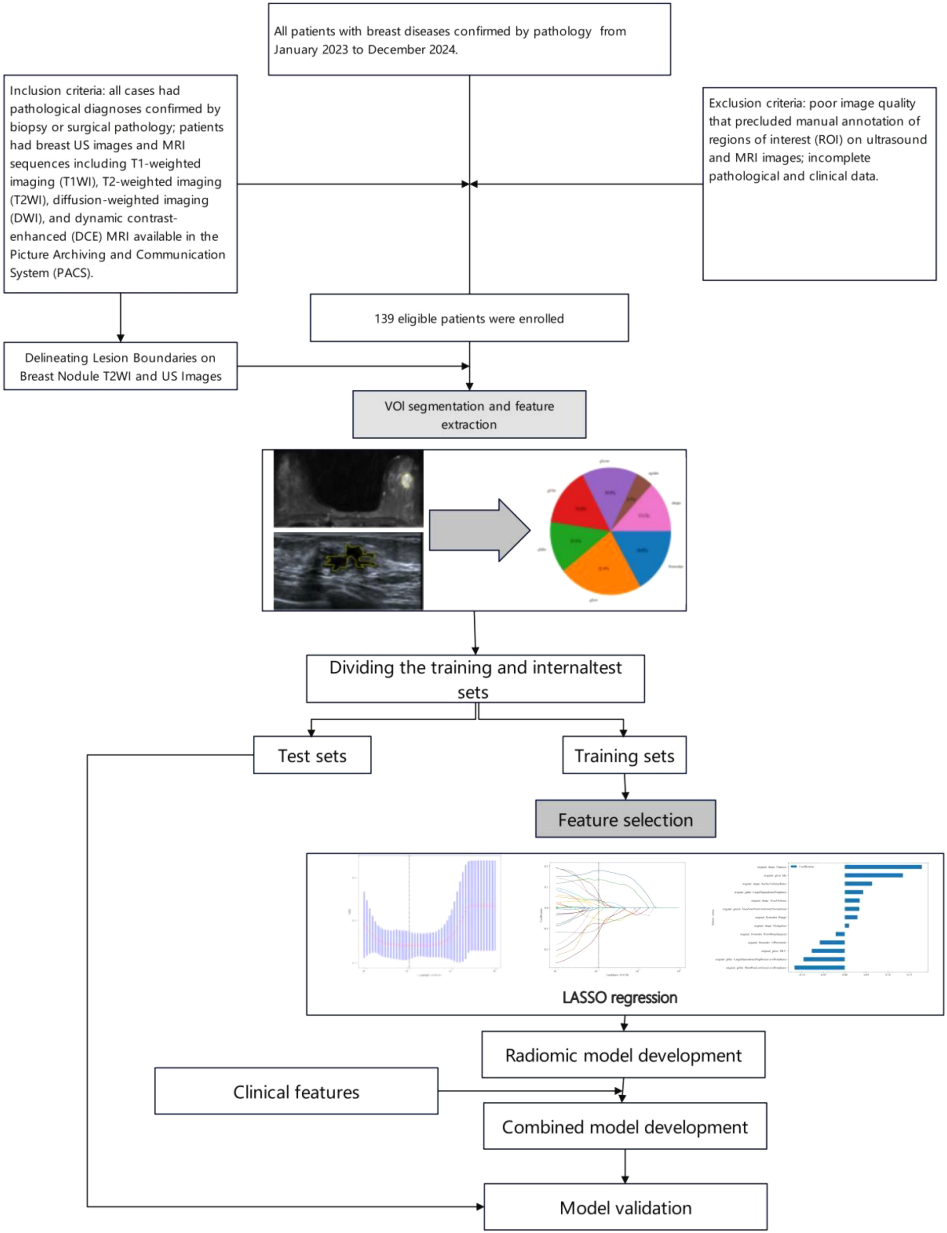
Flow chart of the recruitment pathway for the datasets used in this study.

This study was approved by the hospital ethics committee (Approval No.: 2025CDFSFLYYEC-22). Informed consent was waived due to the retrospective nature of the study. As shown in **Figure 1**, a retrospective collection was made of 139 patients with breast diseases confirmed by pathology at Fuling Hospital Affiliated to Chongqing University from January 2023 to December 2024.All patients underwent both breast ultrasound (US) and contrast-enhanced breast MRI. All patients were female. Among these patients, there were 93 malignant lesions with patient ages ranging from 30 to 85 years, an average age of 54 ± 10 years, and a median age of 53 years; there were 46 benign lesions with patient ages ranging from 20 to 69 years, an average age of 44 ± 10 years, and a median age of 47 years. Benign lesions included 15 fibroadenomas, 12 intraductal papillomas, 3 mastitis cases, 14 adenosis cases, 1 benign phyllodes tumor, and 1 borderline phyllodes tumor; all malignant lesions were breast cancers, including 86 invasive breast cancers, 4 ductal carcinoma *in situ*, 1 invasive micropapillary carcinoma, 1 tubular carcinoma, and 1 intracanalicular papillary carcinoma. Inclusion criteria: all cases had pathological diagnoses confirmed by biopsy or surgical pathology; patients had breast US images and MRI sequences including T1-weighted imaging (T1WI), T2-weighted imaging (T2WI), diffusion-weighted imaging (DWI), and dynamic contrast-enhanced (DCE) MRI available in the Picture Archiving and Communication System (PACS). Exclusion criteria: poor image quality that precluded manual annotation of regions of interest (ROI) on ultrasound and MRI images; incomplete pathological and clinical data. Lesions were randomly divided into training and validation sets in an 8:2 ratio, resulting in a training cohort (n=111) and a test cohort (n=28).

### 1.2 Methods

Patients’ MRI images were acquired from two machines: Siemens Skyra 3.0T MRI and Siemens 1.5T MRI, using dedicated breast coils. Patients were positioned in the prone position. Conventional scans were performed first, including T1-weighted imaging (T1WI), T2-weighted imaging (T2WI), and diffusion-weighted imaging (DWI) sequences. The scanning parameters were as follows: for T1WI, TR 6.5 ms, TE 3.0 ms, slice thickness 3.0 mm; for T2WI, TR 4500 ms, TE 80 ms, slice thickness 4.0 mm. Subsequently, dynamic contrast-enhanced MRI (DCE-MRI) was performed. Contrast enhancement used intravenous injection of gadoteric acid meglumine at a dose of 0.1 mmol/kg body weight, injected at a rate of 2–3 ml/s. A mask scan was performed before contrast injection, followed by continuous enhanced scanning for 8 minutes and 7 seconds, totaling 8 phases, with each dynamic phase lasting 60 seconds. The number of slices was 96, slice thickness = 1.6 mm, flip angle 10 degrees, TR/TE 4.66 ms/1.7 ms, matrix 448x448, FOV 36 cm x 36 cm. The first phase image of DCE T1WI was selected for analysis.Ultrasound images were obtained from Voluson E10 and Logiq E9 (GE Healthcare Systems, USA) color Doppler ultrasound diagnostic equipment.All images will be preprocessed to reduce the impact of differences between devices, and all image voxels will be set to 1mm x 1mm x 1mm.All MRI image analyses in this study were independently conducted by two doctors with more than five years of experience in breast MRI diagnosis, while ultrasound image analyses were independently conducted by two doctors with more than five years of experience in breast ultrasound diagnosis. When disagreements occurred, a senior physician would unify the opinions.

### 1.3 Radiomics analysis

#### 1.3.1 Image segmentation

**Figure 2:**
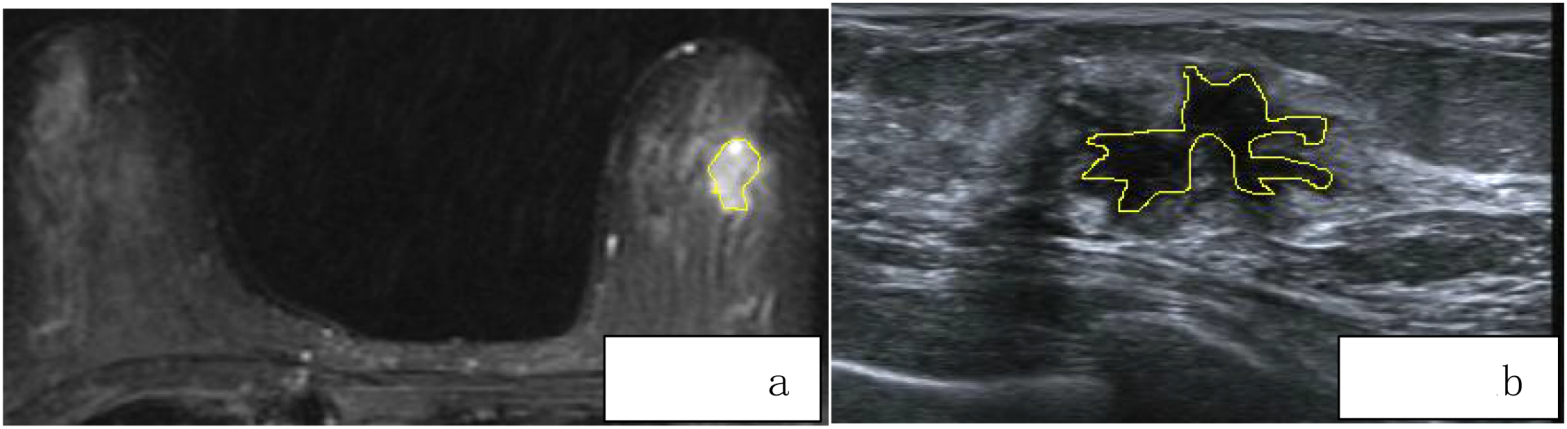
**(a, b)** Delineating Lesion Boundaries on Breast Nodule T2WI and US Images.

For each patient, axial T1-weighted imaging (T1WI), T2-weighted imaging (T2WI), diffusion-weighted imaging (DWI), and the first phase of dynamic contrast-enhanced MRI (DCE-MRI) images were exported in standard DICOM format. A radiologist with 8 years of diagnostic experience used 3D Slicer software to manually delineate regions of interest (ROI) along the tumor margins on the exported images. These segmented images were then used for radiomic feature extraction. Another radiologist with 10 years of experience in imaging diagnosis confirmed the segmented images. The ROI delineation should follow these guidelines:(1)According to the size, shape, and margin characteristics of the lesion, the ROI should be drawn closely along the inner edge of the tumor.(2)Lesion changes such as liquefaction, cystic degeneration, fat necrosis, and necrosis should be included within the ROI region as part of the tumor characteristics.(3)When the lesion boundary is unclear, other sequence images from the same slice should be referenced for accurate delineation (**Figure 2A**). Breast ultrasound images of patients were acquired by a physician with over 5 years of experience in breast ultrasound diagnosis and exported in JPG format. Two ultrasound physicians, one with 5 years of experience and another with 8 years of experience, independently performed manual segmentation along the tumor margins using 3D Slicer software, without prior knowledge of the pathological results (**Figure 2B**).

#### 1.3.2 Feature extraction and selection

**Figure 3:**
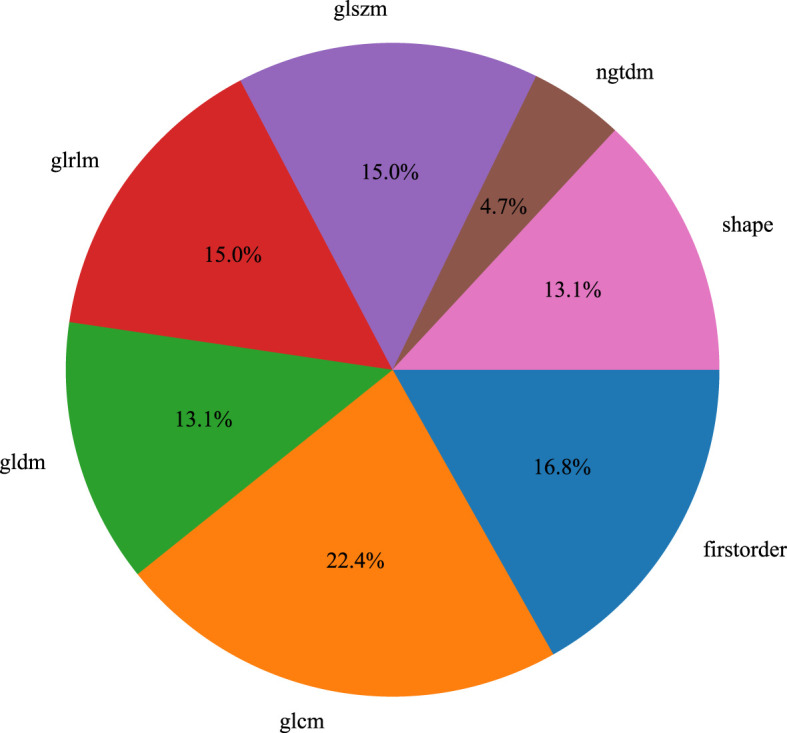
Feature extraction and ratio analysis.

We used the PyRadiomics package in Python to extract radiomic features from the volumes of interest (VOIs) in T1-weighted imaging (T1WI), T2-weighted imaging (T2WI), diffusion-weighted imaging (DWI), dynamic contrast-enhanced MRI (DCE), and ultrasound (US) images (16). For each volume of interest, we extracted a total of 107 radiomic features, which were categorized into three groups: geometric shape, intensity, and texture. Geometric features and intensity features describe the three-dimensional shape characteristics of the VOI and the first-order statistical distribution of voxel intensities within the VOI, respectively, while texture features describe the second-order and higher-order spatial distribution patterns of intensity. The extracted features are as follows: (1) First-order statistics (n = 18), (2) Shape (n = 14), (3) GLCM (n = 24), (4) GLDM (n = 14), (5) GLRLM (n = 16), (6) NGTDM (n = 5), (7) GLSZM (n = 16), **Figure 3**.

Breast cancer physical examination symptoms or positive findings include: palpable nodules or masses in the breast, nipple discharge, asymmetric thickening or masses in the breast, skin changes, axillary masses, and breast pain, among others (17), Therefore, the clinical features we included are as follows::age、maximum diameter、Intra tumor interval、Adipose degeneration、Cystic change、Axillary lymph nodes were enlarged、Deep lobulation、Polytuberous fusion、CA125、CA153、CA199、CEA、Breast pain、Nipple discharge、Breast skin depression、Breast lymph reflux was blocked.

All extracted radiomic features were standardized using z-scores (mean of 0 and standard deviation of 1) to conform to a standard normal distribution. Features that significantly distinguished between benign and malignant breast nodules were selected using a t-test (P < 0.05). Pearson correlation coefficients were calculated, and when the value was less than 0.75, it indicated that the selected features were not correlated. Finally, the least absolute shrinkage and selection operator (LASSO) analysis was used to determine the most useful radiomic features. Clinical features were selected through univariate and multivariate logistic regression analysis.

### 1.4 Construction of radiomic and combined models

We developed radiomic models based on T1-weighted imaging (T1WI), T2-weighted imaging (T2WI), diffusion-weighted imaging (DWI), dynamic contrast-enhanced MRI (DCE), and ultrasound (US) images. The base classifier acts as a feature encoder and has a significant impact on classification (18), Five machine learning algorithms were used: Support Vector Machine (SVM), K-Nearest Neighbor (KNN), Random Forest, Decision Tree, and Extreme Gradient Boosting (XGBoost), to determine the most suitable algorithm for distinguishing between benign and malignant breast nodules. Filtered radiomic features were incorporated into the classifiers and trained on the training set to develop the radiomic models.The results from binary classification served as the reference standard, with benign nodules encoded as 0 and malignant nodules encoded as 1 during classifier training. We obtained the predicted probabilities of benign and malignant nodules as radiomic features. Finally, we developed a combined model using multivariate logistic regression based on both radiomic and clinical features.

### 1.5 Establishment of the nomogram

To simplify the combined model into an easily understandable tool, a nomogram was utilized to create a simplified graphical display. The total points of the nomogram were calculated based on both clinical features and radiomic features.

### 1.6 Statistical analysis

Statistical analysis was performed using SPSS 26.0 (IBM) and R Studio (version 4.3.1). Clinical features were analyzed using the chi-square test or Fisher’s exact test, as they are all categorical variables. In univariate and multivariate logistic regression analyses, clinical features with a p-value < 0.05 were included in the combined model. The differences in radiomic features were assessed using the t-test or Mann-Whitney U test, as they are continuous variables. The statistical significance level was set at a p-value < 0.05.

## 2 Results

### 2.1 Comparison of clinical data

The clinical features were compared (**Table 1**), and we observed no significant differences between the clinical features of the training and internal test sets (p = 0.059–1). The clinical characteristics of patients in the training and validation groups are shown in **Table 2**. The clinical features of patients in both groups indicated that Age, CA199, and Deep lobulation were independent risk factors for distinguishing between benign and malignant breast nodules (P < 0.05).

**Table 1:** Baseline characteristics of benign and malignant breast nodules.

Characteristics	Training set (n=111)	Test set (n=28)	P value
CA125	10.79 ± 3.46	10.92 ± 1.50	0.115
CA153	14.09 ± 9.22	14.82 ± 8.41	0.44
CA199	25.54 ± 0.47	25.54 ± 0.00	1
CEA	2.01 ± 1.92	1.53 ± 0.66	0.455
Age	51.25 ± 11.05	50.79 ± 12.26	0.845
Maximum diameter	23.27 ± 16.05	23.18 ± 15.31	0.811
Intra tumor interval			1
–	105 (94.59)	27 (96.43)	
+	6 (5.41)	1 (3.57)	
Adipose degeneration			0.059
–	68 (61.26)	11 (39.29)	
+	43 (38.74)	17 (60.71)	
Cystic change			0.539
–	42 (37.84)	13 (46.43)	
+	69 (62.16)	15 (53.57)	
Axillary lymph nodes were enlarged			0.935
–	60 (54.05)	16 (57.14)	
+	51 (45.95)	12 (42.86)	
Deep lobulation			1
–	62 (55.86)	16 (57.14)	
+	49 (44.14)	12 (42.86)	
Polytuberous fusion			1
–	91 (81.98)	23 (82.14)	
+	20 (18.02)	5 (17.86)	
Breast pain			0.314
–	104 (93.69)	24 (85.71)	
+	7 (6.31)	4 (14.29)	
Nipple discharge			0.788
–	103 (92.79)	27 (96.43)	
+	8 (7.21)	1 (3.57)	
Breast skin depression			1
–	103 (92.79)	26 (92.86)	
+	8 (7.21)	2 (7.14)	
Breast lymph reflux was blocked			1
–	109 (98.20)	28 (100.00)	
+	2 (1.80)	0	

**Table 2:** Results of univariate and multivariate logistic regression analyses for classifcation of benign and malignant breast nodules.

Characteristics	Univariate analysis			Multivariate analysis		
	OR	95%CI	P value	OR	95%CI	P value
Nipple discharge	6.000e-01	0.181-inf	0.484			
Age	1.017e+00	1.01-inf	0	1.083	1.025-1.143	0.017
CA199	1.027e+00	1.014-inf	0.001	0.739	0.626-0.873	0.003
Maximum diameter	1.043e+00	1.026-inf	0	1.014	0.973-1.057	0.584
CA153	1.051e+00	1.026-inf	0.001	1.028	0.947-1.115	0.578
CA125	1.063e+00	1.03-inf	0.001	1.11	0.994-1.241	0.121
Breast_pain	1.333e+00	0.379-inf	0.706			
CEA	1.621e+00	1.33-inf	0	1.636	0.657-4.076	0.374
Cystic change	2.286e+00	1.486-inf	0.002	2.097	0.664-6.619	0.289
Adipose degeneration	5.143e+00	2.606-inf	0	3.051	0.986-9.44	0.104
Polytuberous fusion	5.667e+00	2.024-inf	0.006	0.84	0.1-7.029	0.893
Axillary lymph nodes were enlarged	6.286e+00	3.219-inf	0	2.732	0.884-8.44	0.143
Deep lobulation	4.800e+01	9.107-inf	0	37.04	5.529-247.894	0.002
Breast lymph reflux was blocked	2.652e+09	0-inf	1			
Intra tumor interval	7.837e+09	0-inf	0.999			
Breast skin depression	3.779e+10	0-inf	1			

e denotes scientific notation format, for example, 1.23e-04 represents 1.23×10^−4^. The term inf indicates infinity and is typically used to denote a value that exceeds the range that can be handled by the computer.

### 2.2 Establishment and validation of radiomic models

**Figure 4:**
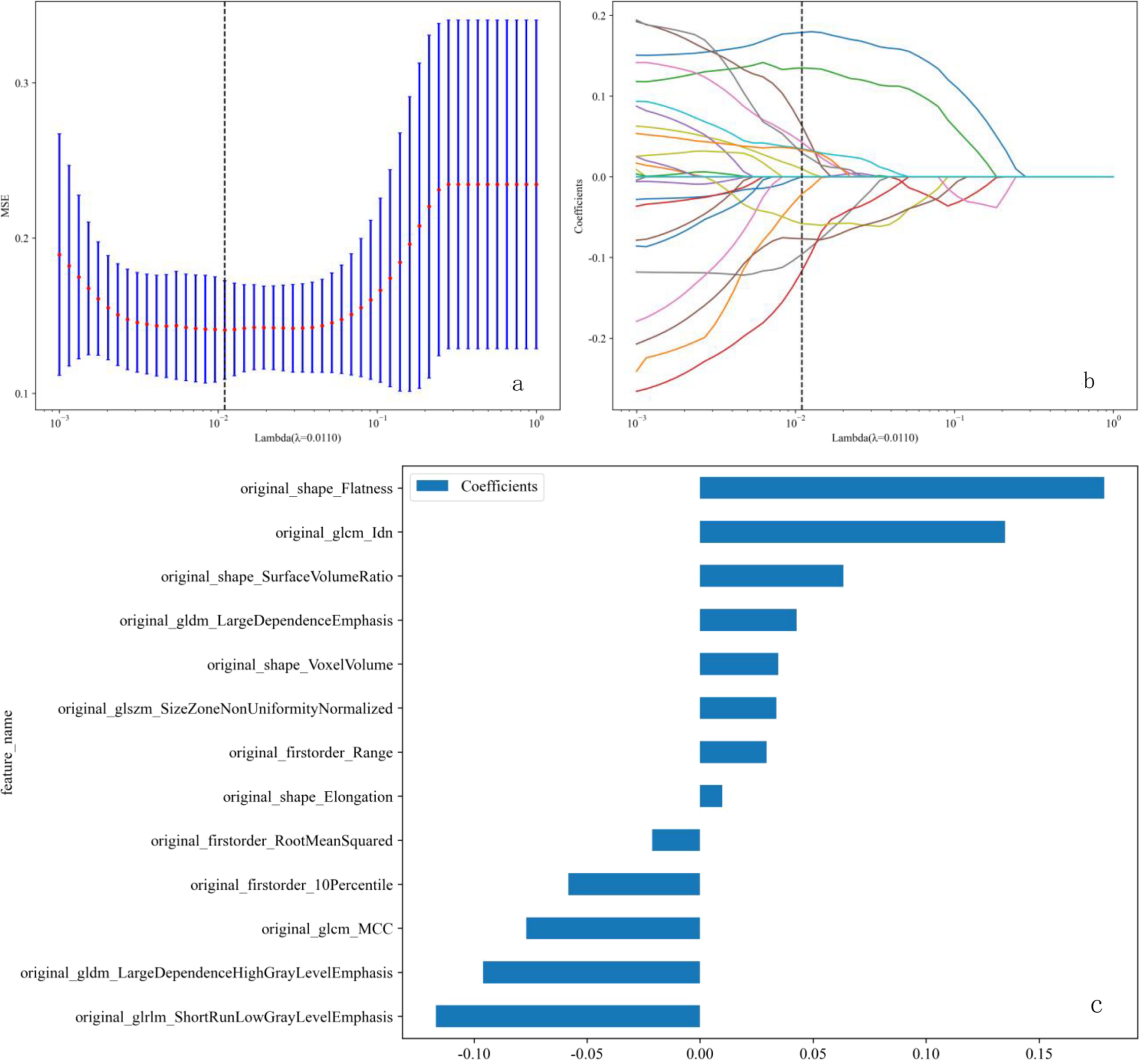
**(a-c)** LASSO regression analysis procedure for the selection of radiomic features. **(a)** The λ parameter in the LASSO model was adjusted using 10-fold cross-validation to achieve the minimum mean square error. The optimal λ value is 0.011, as indicated by the vertical dashed line. **(b)** Representative LASSO coefficient distribution plots. A vertical dashed line is drawn at the value selected after 10-fold cross-validation, based on the coefficient distribution map generated from the λ sequence. The best λ value was used to filter the non-zero coefficients. **(c)** Coefficient values for 13 features. LASSO, least absolute shrinkage and selection operator.

We established five radiomic models to differentiate between benign and malignant breast nodules using T1WI, T2WI, DCE, DWI, and US images. For the T1WI radiomic model, the training set AUC was 0.885 with a sensitivity of 0.865 and specificity of 0.811, while the test set AUC was 0.778 with a sensitivity of 0.632 and specificity of 0.889. For the T2WI radiomic model, the training set AUC was 0.950 with a sensitivity of 0.946 and specificity of 0.865, and the test set AUC was 0.871 with a sensitivity of 0.579 and specificity of 1.000. For the DCE radiomic model, the training set AUC was 0.854 with a sensitivity of 0.784 and specificity of 0.838, and the test set AUC was 0.749 with a sensitivity of 0.368 and specificity of 1.000. For the DWI radiomic model, the training set AUC was 0.878 with a sensitivity of 0.726 and specificity of 0.914, and the test set AUC was 0.763 with a sensitivity of 0.684 and specificity of 0.875. For the US radiomic model, the training set AUC was 0.878 with a sensitivity of 0.703 and specificity of 0.946, and the test set AUC was 0.737 with a sensitivity of 0.737 and specificity of 0.667. The results showed that the T2WI radiomic model had the highest AUC. Through t-tests, Spearman correlation analysis, and LASSO regression analysis, we obtained 13 radiomic features, including 4 shape features, 3 intensity features, and 6 texture features (see **Figures 4a–c**). Logistic Regression (LR) performed best in distinguishing between benign and malignant breast nodules (**Table 3**). Therefore, we selected it as the base classifier for the radiomic model and obtained the radiomic features.

**Table 3:** Performance comparison of different models.

model_name	TASK	AUC	SENS	SPEC
LR	TRAIN	0.950 (0.9006-0.9986)	0.946	0.865
	TEST	0.871 (0.7386 - 1.0000)	0.579	1.000
NaiveBayes	TRAIN	0.909 (0.8438 - 0.9736)	0.824	0.919
	TEST	0.825 (0.6660 - 0.9832)	0.684	0.889
SVM	TRAIN	0.940(0.8879 - 0.9915)	0.838	0.946
	TEST	0.854 (0.7036 - 1.0000)	0.789	0.889
RandomForest	TRAIN	0.957 (0.9095 - 1.0000)	0.932	0.946
	TEST	0.602 (0.3712 - 0.8335)	0.316	0.889
ExtraTrees	TRAIN	0.985 (0.9597 - 1.0000)	0.973	0.946
	TEST	0.784 (0.6072 - 0.9601)	0.737	0.778
XGBoost	TRAIN	0.992(0.9790 - 1.0000)	0.959	0.973
	TEST	0.708(0.4949 - 0.9203)	0.632	0.889
LightGBM	TRAIN	0.841(0.7616 - 0.9203)	0.000	1.000
	TEST	0.778(0.6016 - 0.9539)	0.000	1.000
GradientBoosting	TRAIN	0.991(0.9754 - 1.0000)	0.946	0.973
	TEST	0.749(0.5492 - 0.9478)	0.632	0.889
AdaBoost	TRAIN	0.967(0.9401 - 0.9942)	0.919	0.865
	TEST	0.766(0.5898 - 0.9424)	0.474	1.000
MLP	TRAIN	0.927(0.8740 - 0.9800)	0.824	0.973
	TEST	0.678(0.4635 - 0.8933)	0.579	0.889

### 2.3 Establishment and validation of the combined model

**Figure 5:**
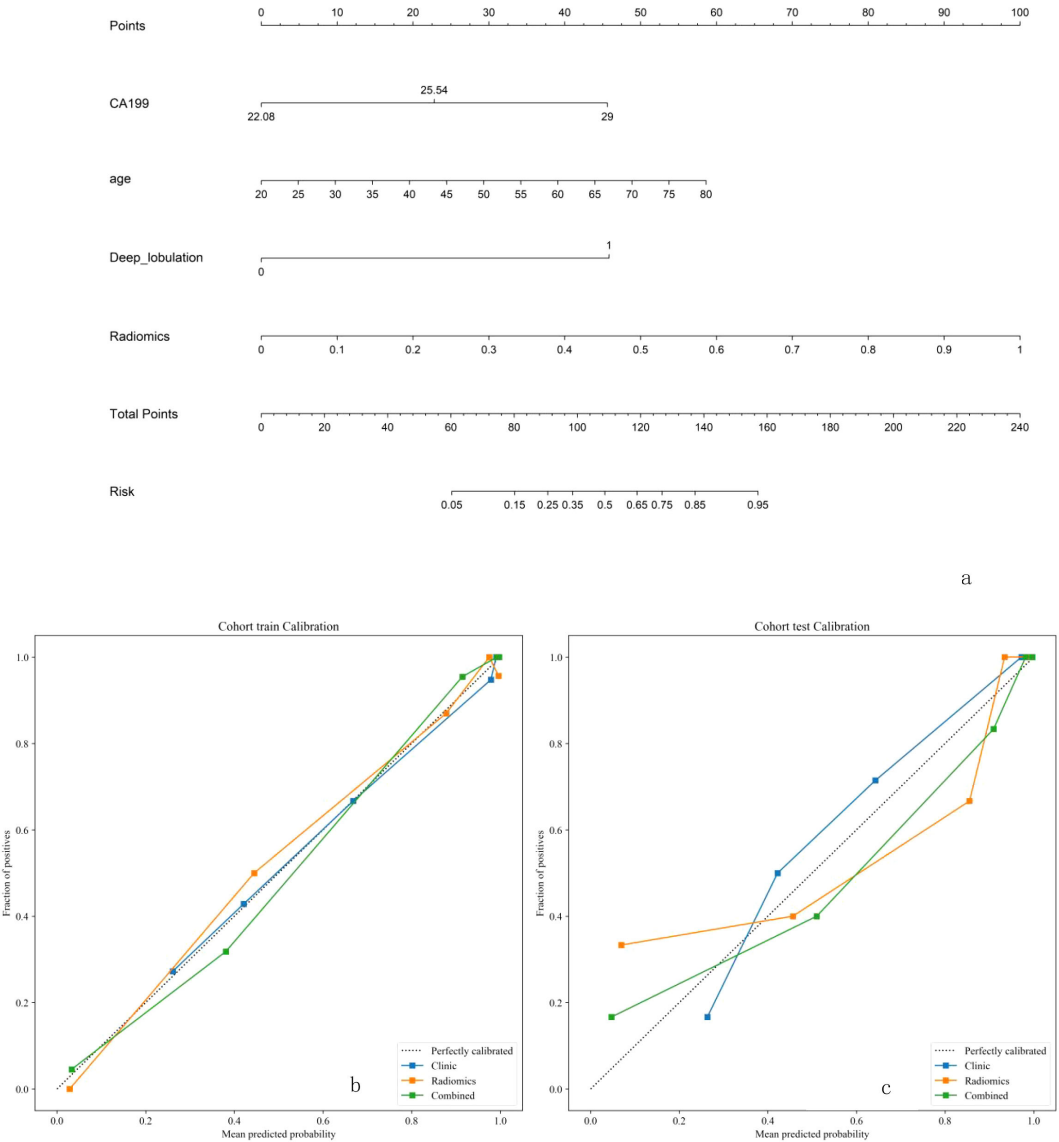
**(a-c)** The combined model for classifcation benign and malignant breast nodules. The calilbration curves of this model in the training and interaltest sets were obtained by resampling 1000 times.The dotted line indicates the ideal ability, and the solid line represents the real ability of the model. The clinic、radiomics、combined model all performed well when the solid line was closer to the dotted line.

Due to the highest AUC of T2WI, we decided to use T2 and clinical data to establish a combined model. Using selected clinical and radiomic features, we developed and visualized a comprehensive model in the form of a nomogram. The AUC values for the comprehensive model on the training set and test set were 0.975 (95% CI = 0.9525 - 0.9978) and 0.942 (95% CI = 0.8612 - 1.0000), respectively. The calibration curves of the nomogram showed acceptable agreement between predictions and actual observations (**Figures 5a–c**).

### 2.4 Comparison of radiomic and combined models

**Figure 6:**
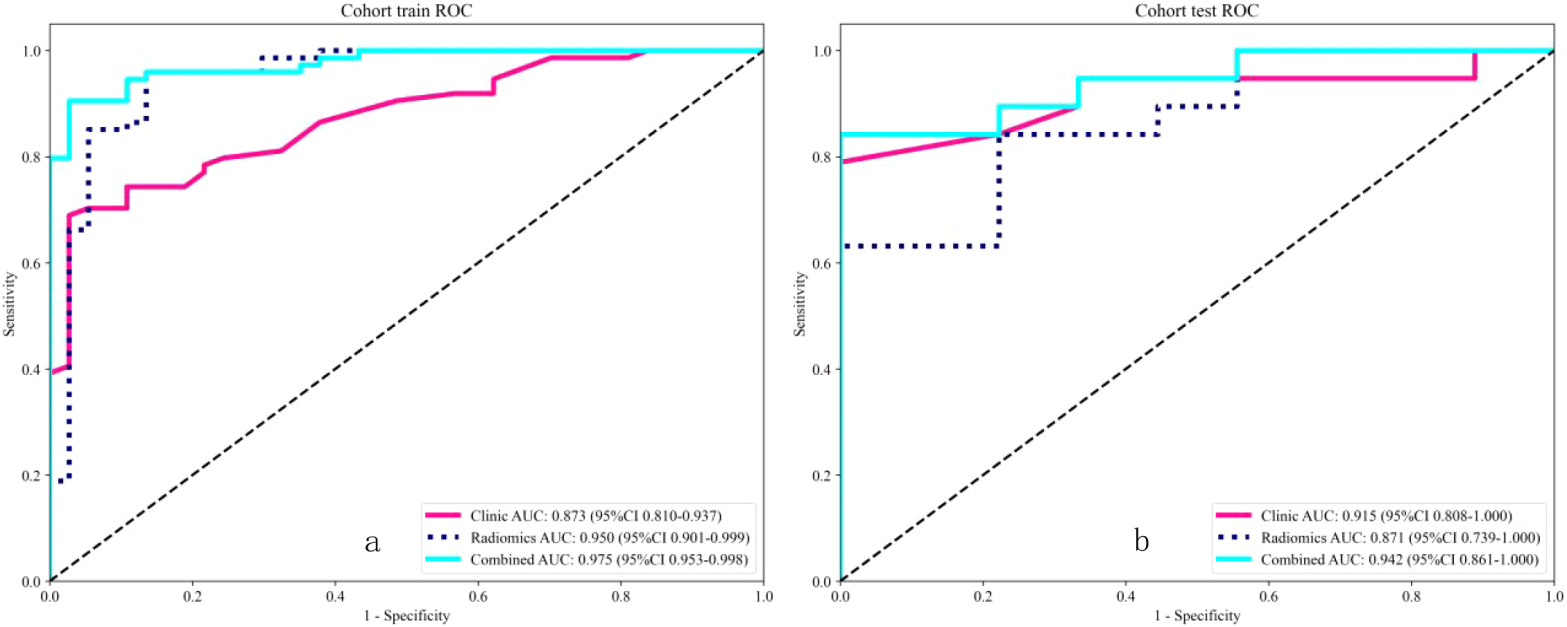
Receiver operating characteristic (ROC) analysis revealed that for the classification of benign and malignant breast nodules, the performance of the combined model performed best in both the training and internal test sets.

The DeLong test showed that the combined model outperformed the radiomic model (**Table 4**). The receiver operating characteristic (ROC) curves for the radiomic and combined models are shown in **Figures 6a, b**. In the training set, the combined model (AUC = 0.975, 95% CI = 0.9525 - 0.9978) was superior to the radiomic model (AUC = 0.950, 95% CI = 0.9006 - 0.9986) and the clinical model (AUC = 0.873, 95% CI = 0.8100 - 0.9369). In the test set, the combined model (AUC = 0.942, 95% CI = 0.8612 - 1.0000) was superior to the radiomic model (AUC = 0.871, 95% CI = 0.7386 - 1.0000) and the clinical model (AUC = 0.915, 95% CI = 0.8083 - 1.0000).

**Table 4:** Performance comparison of the radiomic and combined models.

Model	AUC	SENS	SPEC	Cohort
Clinic	0.873 (0.8100 - 0.9369)	0.676	0.973	train
Radiomic model	0.950 (0.9006 - 0.9986)	0.946	0.865	train
Combined model	0.975 (0.9525 - 0.9978)	0.892	0.973	train
Clinic	0.915 (0.8083 - 1.0000)	0.737	1.000	test
Radiomic model	0.871 (0.7386 - 1.0000)	0.579	1.000	test
Combined model	0.942 (0.8612 - 1.0000)	0.789	1.000	test

*AUC*, area under the receiver operating characteristic curve; *SENS*, sensitivity; *SPEC*, specifcity.

## 3 Discussion

The results of this study indicate that a comprehensive model based on clinical and radiomic features can accurately classify benign and malignant breast nodules, and this model is expected to provide value for clinical decision-making. Breast MRI has advantages such as good soft tissue resolution and no radiation, while ultrasound can provide real-time dynamic images, allowing doctors to immediately observe the structure and blood flow of the breast. Compared with mammography, it has significant advantages in early diagnosis and local staging of breast cancer. According to the NCCN guidelines (19), BI-RADS4 breast lesions have a probability of malignancy ranging from 2% to 95%, and inaccurate visual assessment may lead to incorrect diagnoses. Radiomics can transform medical images into high-throughput mineable data and automatically extract features to supplement clinical indicator estimates for various cancers, including breast cancer (11, 20), thereby compensating for the limitations of visual image assessment. Mudigonda et al. reported that malignant tumors exhibit significantly different gradient and GLCM features compared to benign nodules, especially at tumor boundaries (21), Features derived from GLCM and GLRLM can be used to characterize breast lesions, showing significant differences between benign and malignant lesions (22, 23); Radiological models based on MRI are also helpful in screening, particularly in cases of suspicious BI-RADS 4a or 4b lesions. Based on multi-parametric MRI (mpMRI), Zhang et al. developed a model with an AUC of 0.946, and combining radiomic scores with BI-RADS scores further improved the AUC to 0.975 (24).Han et al. (25)studied 178 breast cancer patients with contralateral BI-RADS4 lesions and concluded that machine learning models based on MRI radiomics could improve the accuracy of evaluating contralateral BI-RADS4 lesions. Therefore, this study aimed to develop radiomic models based on DCE-MRI, DWI, T1WI, T2WI, and US images using algorithms such as LR, NaiveBayes, and SVM to differentiate between benign and malignant breast nodules. Our results, analyzed through ROC curves, Hosmer-Lemeshow tests, and calibration curves, showed that the T2WI model performed best among radiomic models, likely due to its superior display of edema and important lesions. We integrated features from the best-performing radiomic and clinical models to construct a nomogram. A highlight of this study is the development of a combined model incorporating multiple sequences and clinical indicators from different modalities, visualized in the form of a nomogram, making it easier for readers to understand the impact of each factor on classification. However, our study has some limitations. First, it is a retrospective study. Prospective studies are needed to further validate our model. Second, this study included only 139 patients, lacking large-scale research, as reported by Doris Leithner et al. (26), some of our findings may be the result of overfitting when the dataset is small. Future studies should include more patients and standardized protocols to build better predictive models. Finally,Although we have performed resampling preprocessing on all images to reduce the differences between images from different devices, these differences are objectively present. Therefore, we hope that better technologies will emerge in the future to mitigate the impact of differences between devices and improve the reproducibility of imaging studies.

## 4 Conclusion

This study established a classification model for benign and malignant breast nodules based on MRI-T2WI imaging. The comprehensive model performed well in classifying benign and malignant breast nodules and is expected to provide value for clinical decision-making.

## Data Availability

The original contributions presented in the study are included in the article/supplementary material. Further inquiries can be directed to the corresponding author.
